# Some Suggestions for Better Country Roads

**Published:** 1893-02

**Authors:** Sarah Cooper Hewitt


### SOME SUGGESTIONS FOR BETTER COUNTRY ROADS.

By Sarah Cooper Hewitt.

So much has been said about the difficulty of making good country roads without involving a great outlay of money that it seems rather presuming to take a contrary view of the subject, but I think the matter has been much exaggerated, and that in any part of the country where clay, hard-pan, gravel or disintegrated rock can be found it is quite easy to get excellent roads at comparatively little expense. I speak from some practical experience acquired in road-making in a very wild and hilly region of northern New Jersey, where we are accustomed to work out our taxes on thirteen or more miles of public highway, and by adopting the following system we have found it easy to keep the roads in such good condition that they can be driven over at all times with speed, comfort and pleasure.

The proper time to begin work or repairs is in the early spring, just after the frost comes out of the ground, as soon as the roads have thoroughly settled and dried out. Where the road is old, with a good solid bed, the first thing to be done is to cover it all over with a light dressing of gravelly material, and when possible finish it off with a top coating of hard-pan; but avoid putting it on too thickly, lest the going become heavy, as it is slow to dry out. Loam is worse than useless, because it never packs properly, and makes mud. Care must be taken to raise the road up toward the centre, and give it a slight elevation at the crown, but only just enough to shed the water on either side into the gutters. On a level, straight road the crown should only be slightly convex, for rounding up a narrow road in the middle is objectionable, insomuch as it has the immediate effect of forcing wheels of vehicles to run always in the same line and wear away the new material into deep ruts that quickly become watercourses for the wash of the next rain, and assist the rapid destruction of the road by preventing the water from reaching the gutters As fast as the new material can be laid on, it should be very carefully raked over to remove all the large stones and as many of the smaller ones as possible; for where this precaution is neglected until the stuff packs down hard, which happens in a few days, the stones become so firmly embedded that they are not only difficult to remove, but soon cause the road to wear in humps and bumps, and later in the season, during the dry weather, they work up continually and become a source of annoyance and danger. These stones should never be left in piles along the road-side, to be driven into or scattered about by mischievous boys or stray cattle, but carted immediately away and dumped out of sight. The gutters should then be carefully cleaned by removing from them all deposits of mud, decayed leaves or branches which have collected there during the winter months; never allow this stuff to be heedlessly thrown along the edges of the gutters or on the banks above them, for the first rains will surely wash all back to its old place, and the work has to be done over again. It is even worse to spread it out on the road, according to the common but mistaken practice of many road-masters, since decayed material can never pack properly, and always tends to make mud in wet and dust in dry weather. Another practical reason in favor of keeping the gutters free just after the roads are first repaired, is that when the heavy spring and summer showers have washed away the greater portion of the good new coating, it is at once caught and retained in the gutters, ready to be used the first time the road needs patching, when a man or two can quickly put it in first-class order by simply shovelling back the material into its old place again and then raking it over.

In the early spring, while the gravel or hard-pan is still sticky or heavy, the process of drying out and packing down can be greatly accelerated by keeping some one raking over the road to level off and smooth down the ruts as fast as they are made by the cutting in of the wheels, instead of allowing them to wear down deeper and deeper and to furnish sure channels for the wash of water which adds to the difficulty and expense of repairing. This method is quicker, easier, and far more economical than rolling, which requires a pair of horses, and it is surprising how great a distance a smart worker can get over and put in perfect order during a day. By repeating this raking once or twice, according to the condition of the road and the amount of travel upon it, the surface will pack down quite as hard and even as that of a park road.

Whenever a mud-puddle appears, owing to some slight depression, in a place so shaded by trees or shadows of hills that the sun loses its power, it should never be left to dry out slowly, day after day, giving the wheels a chance to cut into it more deeply, but as soon as discovered a cart-load of good dry gravel should at once be dumped into it, then raked off, tamped down, and perhaps reraked until it packs hard and smooth.

Unfortunately farmers and road-masters have a fixed idea that the one way to prevent hills, long and short, from washing, is to heap upon them quantities of those original tumular obstructions known indifferently as "thank-you-ma'ams," "breaks," or "hummocks," and the number they can squeeze in upon a single hill is positively astonishing. I remember one hill, less than a quarter of a mile long, where I counted once as many as ten of these horse-killers and carriage destroyers, yet in spite of these clumsy precautions the hill was always in bad order, and horses obliged to walk up it the whole way. Now, however, since the "breaks" have been removed and replaced by culverts, and the road properly graded and rounded at the crown, horses can trot easily either up or down, and it costs very little to keep the hill in good repair. Of course much eloquence, tact and flattery must be expended to bring about the desirable result of inducing road-masters to abandon their most cherished belief, but in the interest of good roads it is well worth the effort on the part of everybody. When "breaks" are done away with, the crown should merely be raised somewhat higher than on a level road, giving it a good pitch to the gutters, which in this case must be sufficiently wide and deep to contain the large amount of running water that often accumulates so rapidly on a hill during a heavy shower as to resemble a small torrent. At certain intervals, therefore, where too large a body of water would collect for the capacity of the gutters, instead of the antiquated "break," a culvert must be introduced, which will afford the means of carrying off the overflow into the adjoining woods or fields.

This question of culverts is really quite an important one, since they bear almost the same relations to roads that key-stones do to arches. Culverts made by putting together jointed cement or glazed earthen-ware pipes are the most satisfactory, being easier handled and comparatively inexpensive, and when laid a certain distance below the surface, run little or no danger of being broken. But to obviate this they should be laid diagonally across the road, which prevents the weight of wagons from bearing upon them all at once, and also gives them better fall. An eight or twelve inch pipe will carry off a large amount of water, but where the volume increases to the size of a small stream, a twelve to fifteen inch pipe will be found to work admirably. On long hills it is advisable to lay some eight inch pipes at reasonable distances apart, dividing up the gutters into short sections instead of giving them a free flow down the entire length of hill, and attempting to have the mass of water carried off by means of a large pipe introduced at the bottom, for in case of stoppage at this point, the road must necessarily be cut out. Shortening the length of gutters means diminishing the water's force and power to wash away material from banks, or to cut so deeply into the road edges. The difference in the cost of small pipes over a large one is not only exceedingly slight, but the little extra expense involved through putting them in is more than saved in one season alone. A short, steep hill needs oniy a single pipe placed near the foot of the hill. These culverts have one advantage over all others, namely, a concave bottom with a smooth glazed surface, which allows the water to rush through so freely that it carries all obstructions before it, and permits no rubbish to choke up the pipes. These require no further attention than a slight examination every spring to see if the frost has cracked a joint, or the ubiquitous country boy has taken it upon himself to stop up the opening by stuffing small stones into it.

When carefully built, stone culverts are not bad, but they are expensive to make well, and, as a rule, their sides are laid up so carelessly in dry walls of such small-sized stones that they are liable to upheave and be thrown down by frost. Moreover, the flat stones laid across the top are often to badly dressed and fitted together that the gravel covering them keeps sifting through the cracks, filling up the culvert and exposing the holes on top, which are either chinked up with cobble-stones or left bare until some horse gets hurt and a row is made, with the only result that more earth is spread over, and the same process is kept up *ad infinitum.* Left entirely to himself, the native road-master prefers a more primitive culvert of his own make, which has the enormous merit in his eyes of being cheap, quick and easy of construction. His method, delightful in its simplicity, consists in digging a trench across the road, and bridging it over with a few split green chestnut rails cut by the road-side, which are afterwards covered with earth or sod heaped above the level of the road in such a manner as to make a disagreeable "break." Besides its liability to become choked and useless, this sort of culvert is particularly objectionable because it is always neglected and forgotten, being left to rot, until at last some horse's foot crashes through it, and the driver may consider himself lucky if the animal escapes with nothing worse than a slight wrench or scratch. During harvest, when it is almost impossible to get men to do any continuous work not connected with farming, to save time we are sometimes obliged to put in a temporary box culvert, made of planks nailed together like a long narrow box open at both ends. These culverts are a slight improvement on the local ones made from chestnut rails, inasmuch as, being quite flat on top, they do not destroy the road's level surface; but, unless care is taken to have them made of oaken planks, they rot out even more quickly than the others.

It is naturally a simpler matter to keep in good order those roads that have been constructed in the first place upon correct principles; but the so-called "old Revolutionary roads" have such a characteristic way of twisting and winding about or climbing over the highest points of hills to avoid ledges and bowlders that they require an entirely different sort of treatment, and can only be improved by slowly and patiently straightening them out through blasting and by removing all obstructions. Any attempt to improve these roads by covering up their rocks and ledges is worse than useless, for the earth cannot hold for any length of time on their hard smooth surfaces, consequently a season's rains are certain to wash away every bit of covering from the road-bed, leaving the stones more exposed than ever, from which doubtless arises the popular tradition throughout the country that stones actually grow. In spite of many repairs, year after year, small stones would keep working up, and the only way we found to prevent this was, for several seasons, to keep a couple of boys taking out each stone with a pick or crowbar as fast as it appeared, filling up the hole afterwards with the best gravel or hard-pan, tamping it down with a shovel, and then raking it over. In this way a smooth firm road-bed was finally secured to work upon, making all subsequent repairs light and durable. The tops of pointed rocks that stuck up were either knocked off with a sledge-hammer or blown up with a charge of powder, and all obstructions in the gutters removed in the same way, the sharp sudden turns in the road modified and rounded off, and the crown lowered to its proper height. It is extraordinary what inexhaustible patience and wonderful constitutions our ancestors must have possessed to enable them to jolt contentedly, day after day, over the same old obstacles, without its seemingly occurring to them to make any effort to remove the obstructions; and on a really bad road how one longs for similar philosophy and such admirable backbone!

Another excellent precaution, but one rarely attended to, is the cutting away of brush and weed growth along the road, particularly around the inside of a sharp turn or bend, in order to enable one to see if any traps are coming from the opposite direction; for in these days of fast driving, when every man imagines he owns a trotter, a narrow winding road becomes dangerous unless means are provided to get out of the way of the "old mare" as she tears around the corner. Brush when not cut down every season encroaches with incredible rapidity, and spreads so fast into the gutters that if neglected it narrows the roads perceptibly, and the same thing is true of weeds and grass, although in a lesser degree; they also should be removed at least once every year. Not, however, by the following reprehensible process, which finds great favor in the eyes of road-masters and farmers, and consists in sending out the "boy" with a yoke of oxen or the "hired man" with a team to run a couple of furrows up and down the gutters with a plough, thereby loosening but not destroying all this vegetable growth, which is then either left to obstruct the gutters with useless ridges or flung out over the road to pack down in all sorts of unequal masses.

The introduction of broad tires upon all farm wagons and carts adapted for heavy draught purposes alone would do much to improve roads, since half the trouble seems to arise from heavy loads carting over country roads at seasons of the year when the ground is soft. At Tuxedo, where all draught wagons are prohibited an entry unless furnished with broad-tired wheels, the tremendous advantage over the ordinary tires has been plainly proved, for there, even when the roads are softest and at their worst, they never cut up through the constant carting of heavy loads of brick, stone or lumber over them; for the tires, by being so broad that they cannot cut in and hence track in the same place, act somewhat like rollers in keeping the roads hard and smooth. So much might be accomplished in this way if everyone living in the country, when buying a farm wagon or cart, would not only make a point of getting one with broad tires, but would at the same time exert their influence to that effect with their friends and neighbors. For could the merit of these tires as road-improvers once become known throughout the country, public spirit alone would cause their use to become general, and much of the present trouble arising from the deep rutty condition of the roads would cease as if by magic.—*Harper's.*

